# Publisher’s Note

**DOI:** 10.1080/21655979.2024.2326361

**Published:** 2024-03-01

Due to a publisher error, a retraction notice was incorrectly issued on the article ‘miR-148-3p inhibits gastric cancer cell malignant phenotypes and chemotherapy resistance by targeting Bcl2’, https://doi.org/10.1080/21655979.2021.2005742. We have now removed the retraction notice.

We apologise to the authors for this error.

